# Monitoring the efficacy of intraarticular infliximab by musculoskeletal ultrasound and juvenile arthritis disease acitivty score (JADAS) in JIA patients – single center experience

**DOI:** 10.1186/1546-0096-12-S1-P22

**Published:** 2014-09-17

**Authors:** Mandica Vidovic, Lovro Lamot, Marija Perica, Lana Tambic Bukovac, Miroslav Harjacek

**Affiliations:** 1 Pediatric and Adolescent Rheumatology, Children´s Hospital Srebrnjak , Croatia; 2 University of Zagreb School of Medicine, zagreb, Croatia

## Introduction

Pediatric rheumatologists use musculoskeletal ultrasound (MSUS) in everyday practice as a simple, painless and inexpensive tool for detecting synovitis. Along with MSUS, Juvenile Arthritis Disease Activity Score (JADAS) was proven to be valid for clinical assessment. In JIA patients with mono- or oligoarthritis who are inadequately responding to conventional therapy, but do not meet criteria for biological therapy, intraarticular infliximab could be therapy of choice. Since this therapeutic option is not routinely used, there is a need for a continuous follow-up, in which MSUS and JADAS could have great value.

## Objectives

To assess the efficacy of intraarticular infliximab injections in patients with juvenile idiopathic arthritis (JIA) using MSUS and juvenile arthritis disease activity score (JADAS).

## Methods

IA infliximab was administered in 22 joints of 14 patients diagnosed with JIA according to ILAR classification criteria. All patients received first and second line therapy (NSAID, DMARD, corticosteroids systemic and IA). None of the patients fulfilled criteria for treatment with biologic therapeutics, but were resistant to DMARD’s. Intraarticular infliximab (25 mg or 50 mg per joint) was administered.

The patients were monitored by monthly assessment using JADAS (number of active joints, pain assessed by patient/parent an physician (VAS), ESR) and MSUS. The MSUS assessment included Omeract semiquantitative grades (0–3 grades) for both B-mode and Power-Doppler (PD) and 12 patients were examined using 3D/4D US. We used paired samples T-test for comparing JADAS before and after the treatment.

## Results

At the point of IA injections all 22 joints showed grade 2-3 synovitis in B mode and increased PD signal (2-3/3). The mean value of JADAS was 17.31 (± 2.78). At the end of the follow-up period (mean time 7.07 months, range 6-10 months) the mean value of JADAS was 5.66 (± 3.52). There was also improvement in MSUS with 0-1 grade synovitis without effusion in B mode and PD signal decreased to 0-1/3. The deference in JADAS was statistically significant (p< 0.001). However, 3/14 patients subsequently flared (mean time 6-8 months) and fulfilled criteria for systemic biologic therapy. Two of those three patients received lower IA dose of infliximab (25 mg per joint).

## Conclusion

IA infliximab should still be considered as a therapy option in selected children with therapy resistant, isolated mono/oligoarticular JIA. The effect of IA therapy could be easily monitored both by MSUS and JADAS.

## Disclosure of interest

None declared.

